# Shift handover quality in Saudi critical care units: determinants from nurses’ perspectives

**DOI:** 10.1186/s12912-023-01348-z

**Published:** 2023-05-31

**Authors:** Ebtsam Aly Abou Hashish, Atheer Ahmed Asiri, Yara Khaled Alnajjar

**Affiliations:** 1 College of Nursing, King Saud bin Abdul-Aziz University for Health Sciences, Jeddah, Saudi Arabia; 2 King Abdullah International Medical Research Center, Jeddah, Saudi Arabia; 3 Faculty of Nursing, Alexandria University, Alexandria, Egypt

**Keywords:** Handover quality, Critical care units, Nurses, Safety

## Abstract

**Background:**

Nurses’ effective handover communication is vital for patient safety and quality of care. Few studies have empirically tested how certain factors influence the quality of handover in the Saudi context.

**Methods:**

A descriptive correlational design was used with a convenience sample of all nurses (N = 201) working in Saudi hospital CCUs in 2022. Demographics and handover quality instruments were used to collect the necessary data in addition to two open-ended questions that asked about perceived barriers and facilitators to handover. The analysis was conducted using descriptive statistics and regression analysis.

**Results:**

The majority of nurses reported good-quality handover. The regression analysis showed that staffing, cognitive capacity, the focus of attention, relationships, and safety climate factors contributed positively to the variance of handover quality. In contrast, intrusions, distractions, anxiety, time stress, and acute and chronic fatigue factors negatively affected the prediction of handover quality (p < 0.05). Nurses added types of shifts and languages as barriers to handover while emphasizing training and the use of standardized tools for handover as facilitators.

**Conclusion and recommendations:**

Nursing handover is a multidimensional phenomenon. By understanding the determinants that contribute to or hinder handover quality, it is possible to develop targeted interventions aimed at improving communication and the quality of shift handover in CCUs. The current study’s findings highlight the need for nurses to work in a more supportive environment, receive better training, and follow a standardized handover protocol. Additionally, nurse managers should pay more attention to nurses’ well-being to control or mitigate the effect of psychological precursors on the quality of nurses’ handover. Future research should investigate handover practices and outcomes on units that have both good and bad practice environments.

## Introduction

Nurses are present at patients’ bedsides 24 h a day, seven days a week, and they interact with physicians, pharmacists, families, and other health care team members on a daily basis. They are important for timely coordination and communication of the patient’s status to the team [1]. Nurses’ handover is situated within a 24-hour cycle of clinical care in which the nursing, medical, and technical knowledge relevant to each patient needs to be transferred seamlessly between off-going and incoming nurses as they work to maintain safety. Organizations such as the World Health Organization (WHO) and the Agency for Healthcare Research and Quality (AHRQ) have identified communication during clinical handover as a priority to ensure patient safety [1, 2]. The Joint Commission International (JCI) estimated that communication failure during clinical handover was responsible for health care errors and designated efficient communication, including clinical handover, as one of its primary international patient safety goals [3].

The Australian Medical Association (AMA) defines handover as “the transfer of information, professional responsibility, and accountability for some or all aspects of care for the patient, or group of patients, to a person or professional group on a temporary or permanent basis” [4]. Nurse-to-nurse shift handover communication is a crucial information exchange that occurs at shift change to ensure that arriving nurses have all the information they need to take responsibility for their patients and provide high-quality and safe care [5]. The primary goal of shift handover communication is to ensure patient care continuity by providing incoming nurses with the information required to effectively care for assigned patients, such as assessment data, health and safety issues, care delivered, and required care (National Clinical Effectiveness Committee, NCEC) [6]. Handover benefits the organization, the staff, and the patient. Furthermore, handover facilitates communication between nurses and other health care providers about the patient’s health, care plan, and progress as well as informing patients about their care and encouraging patients’ participation in health care. Additionally, it enhances care safety, including medication communication, saves time, allows nursing leaders to model behaviors and share experiences, and assists in student and new staff orientation [5, 7–10]. However, many determinants could affect the quality of nurses’ handover. Our study aims to investigate these determinants.

## Background

The quality of nurses’ handover refers to a nurse-to-nurse communication process that includes the timely and organized exchange of information regarding patient care and assessment to ensure a shared understanding by providing opportunities for questions and clarification [10]. The literature contains a variety of suggestions for procedures and content for a good handover that affect its quality [2]. For instance, handover can occur between nurses at the start and end of shift change and takes between 18 and 50 min to complete according to acuity of illness, clinical specialty, number of patients, time of day, and method of handover communication [8, 10, 11]. Handover delivery can take place bedside, in the office, or in the staff room and typically includes the handing over of patient care information such as the patient’s name, age, diagnosis, and a variety of other information pertaining to the patient and changes in their care, as well as the nurses’ duty of care and responsibility for the next shift [10, 11].

Nurses can use a handwritten or computer-generated handover tool that includes all patients on the unit with complete information for each patient to facilitate its execution [2, 12]. Among the standardized communication tools used for facilitating effective handover is the ISBAR/ISBARQ tool, which refers to “Identify/Introduction, Situation, Background, Assessment, Recommendation, Questions, and Feedback” to emphasize details or obtain a holistic understanding of health care professionals, patients, and family concerns [2, 8]. ISBAR/ISBARQ has grown in popularity around the world to assist health professionals in accelerating cross-disciplinary communication by creating a common information structure that allows nurses to be more focused and spend less time during handovers [2, 8, 13, 14].

### Determinants of handover quality

A variety of determinants influence the quality of the handover throughout its process [5, 7, 8]. So, in this study, we propose that many interconnected factors, such as positive factors, negative factors, barriers, facilitators, or other individual factors, act as determinants and affect the quality of handover [8, 10–12]. Leadership, relationships, staffing, workload, cognitive work, interruptions, stresses, anxiety, fatigue, time pressure, the safety climate, technology, teamwork, the handover format, shift overlap, and staff experience are examples of such factors [10–12]. Moreover, barriers to nurses’ handover may include a lack of communication skills, a lack of diligence in completing handovers or patient records, a lack of standardization, language barriers, insufficient technological support, distractions, a high background noise level, and a lack of a designated time and space [10–14].

### Problem statement

A review of the literature revealed international research on handover in general nursing but little research on critical care nursing [14, 15]. The focus of several studies was on shift-change reports and patient transfers in the general ward [5, 8]. Data in the critical care setting are sparse, and few quantitative nursing studies have been published that investigate the factors that influence the quality of critical care nurse-to-nurse shift handover [14]. The unique environment of CCUs, where there is a high degree of patient unpredictability, increased patient acuity, and complex care procedures, can create challenges for high-quality handover communication among nurses [16]. Given that effective handover in the CCU is even more complex than handover during shift changes or at the bedside on general wards [14, 15], the quality of handover in critical care units needs more investigation [15]. Studies in Saudi Arabia have been limited to investigating barriers to nurse‒patient communication [17, 18]. To the best of our knowledge, there are few Saudi studies of nurses’ handover quality in CCUs. Such studies could be useful for identifying quality and safety improvement areas in handovers as a basis for future interventions in this setting. Therefore, the current study aims to investigate the determinants that influence the quality of nurses’ handover.

### Significance of the study

For many years, international studies have focused on effective communication during shift-to-shift nursing handovers because the accuracy of the information communicated directly impacts patient safety and the quality of care [2, 13]. However, the literature suggests that communication between health care providers is often subject to failure, resulting in negative consequences for patients, staff, and health care organizations [10]. Handover is characterized as a vulnerable and high-risk stage in the care process. Incomplete or inaccurate information, omissions, or misinformation provided during handover communication can lead to a breakdown in communication, uncertainty, and false assumptions by nurses, which can have a direct impact on patient safety [2, 8, 14]. More specifically, ineffective, or poor-quality nurse-to-nurse handover communication in specific care units, such as critical care units, can have a detrimental impact on patients, staff, and health care organizations [2, 15]. Poor handover communication can result in incorrect treatment, delayed medical diagnosis, potentially fatal side effects, death, disability, prescription errors, falls, patient complaints, longer hospital stays, and medication errors (missed or double doses of medications). Misinformation or omissions in the handover between nurses also cause problems, such as disease outbreaks, if nurses enter isolation rooms without realizing they should be wearing protective gear. Additionally, handover communication failures may lead to frustration and anger from both patients and their families when nurses are unable to provide information about diagnostic tests and care plans [8, 19, 20]. Nurses’ perceptions of their communication skills inevitably impact their ability to perform clinical handover [8]. Therefore, investigating the determinants that affect nurses’ handover in each care context especially from nurses’ perspectives is vital to enhance the quality of the handover process and improve patient safety, continuity of care, and staff and organizational results [8, 10, 11, 16].

### Aim of the study

This study aimed to investigate the determinants (positive factors, negative factors, facilitators, and barriers) that influence the quality of nurses’ handover in Saudi critical care units from the perspective of nurses. Further aim was to determine how such factors may be correlated with nurses’ handover quality.

**Research questions**:

- What factors (positive factors, negative factors) influence the quality of nurse handover in critical care units?
- What are the facilitators and barriers that may influence the quality of the handover from the participants’ perspectives?

## Methods

### Research design and setting

**Fig. 1:**
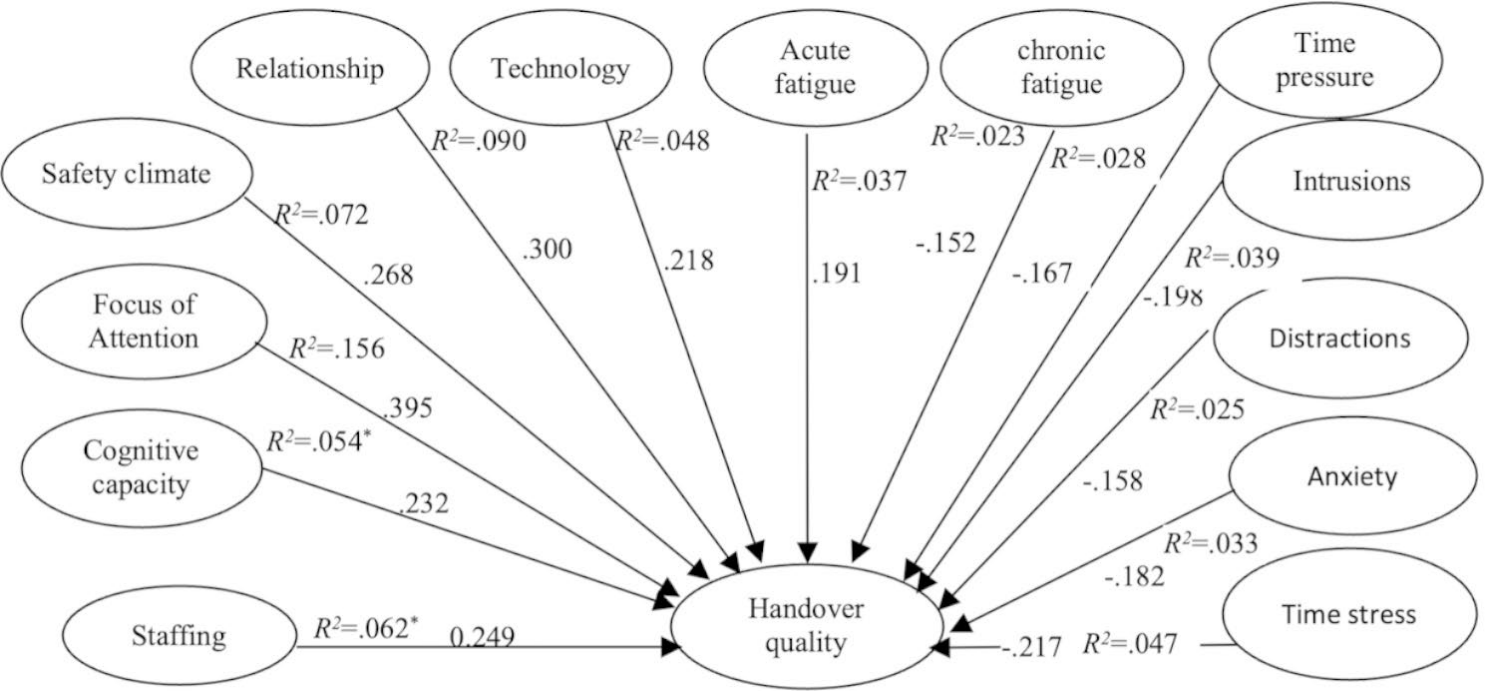
Positive and negative determinants of handover quality

This was a descriptive-correlational research design because it was assumed that many factors would significantly affect nurses’ quality of handover (Fig. 1). The following study hypothesis was postulated:

(H1): There is a significant effect of different factors on the quality of handover.

The study was conducted in all critical care units at King Khalid Hospital (KKH), which is affiliated with King Abdul-Aziz Medical City-Jeddah (KAMC-J) and National Guard Health Affairs Saudi Arabia. The critical care setting included ten units with a capacity of 95 beds.

### Participants and sample

The study included all nurses who were trained and working in the CCUs (N = 208). A purposive sampling technique was applied to recruit eligible nurses based on the inclusion criteria. Nurses who were working in critical care units on day and/or night shifts, had been in their clinical practice area for at least 6 months, and agreed to take part in this study (n = 204) were eligible participants. Exclusion criteria are newly hired nurses who had worked at KAMC-J for less than six months, who are worked one permanent shift and did not involve or take roles in handover, and nurses who worked in general units. Only two nurses are excluded based on these criteria. The sampling frame for each unit was identified from the nurse managers. The sample size was determined using the Raosoft sample size calculator with the following parameters: population size margin error 5, 95% confidence interval (CI), and significance level p ≤ 0.05. Thus, the minimum recommended sample size was 129. To attain the desired sample size, questionnaires were given to eligible nurses working in the CCUs. Of these, 201 nurses completed the questionnaires and returned them, which met the number for the target sample and yielded a response rate of 98.5% of the eligible participants.

### Instruments and measurements

In this study, we used a self-administered questionnaire to collect the data. It included three parts.

#### Part I: Sociodemographic data

This part was developed by the researchers. It aimed to assess nurses’ demographics and work characteristics, such as age, gender, nationality, educational level, working unit, years of experience, the last day of receiving shift handover, and the roles played in handover as outgoing nurses (giving handover), incoming nurses (receiving handover), and varied (both) roles as well as the working shift. Responses were presented using frequencies and percentages.

#### Part 2: quality of handover questionnaire

The quality of handover questionnaire created by Thomson [10] was adapted for this study with author permission. It is a multidimensional tool collected from several studies to determine the factors that affect handover quality. Thompson constructed a questionnaire to assess the factors that affect handover quality in the emergency unit. We adapted the questionnaire for use in CCUs by replacing the word emergency with CCU. Based on expert opinions of its validity, experts suggested replacing the question about date of birth with age (in the demographic section) and removing one question about triage flow because it was more applicable to the emergency unit. Therefore, the final tool included 17 dimensions with a total of 55 items or questions as follows: overall handover quality (1 item), staffing (1 item), intrusions (3 items), distractions (3 items), cognitive capacity (1 item), focus of attention (1 item), anxiety (5 items), time stress (8 items), time pressure (5 items), acute fatigue (5 items), chronic fatigue (5 items), relationships (3 items), safety climate (7 items), technology (2 items), face-to-face communication (2 items), handover tools (2 items), and nurse experience (1 item). Participants’ responses were measured using a variety of response formats, including yes and no, three- and five-point Likert scales of agreement, and multiple-choice formats.

#### Part 3: Factors affecting handover quality from participants’ perspectives

This section was created by the researchers and consisted of two open-ended questions asking participants about the factors that affect the quality of handover from their perspective. The responses of nurses to these questions were analyzed and were configured and arranged using frequency and percentages.

1. What facilitators can affect the quality of handover in your unit?
2. What are the barriers to the quality of nurses’ handover?

### Validity and reliability

Since all participants were university graduates with high proficiency in English, the questionnaire was administered in English. A jury of five experts tested the questionnaire’s three components for content validity. They were asked to evaluate the questionnaire based on item relevance, comprehensiveness, and comprehension. The questionnaire was found to be a relevant, comprehensive, and clear tool, but the experts recommended replacing the question about date of birth with age (in the demographic section) and removing one question about triage flow because it was more applicable to the emergency unit. The content validity of the questionnaire was evaluated using an index based on the rating agreement of five experts, and the content validity index (CVI) was 87.80, proving that it is valid. Additionally, a pilot study was conducted on a sample of nurses (5%) for face validity which resulted in no change in the questionnaire. The reliability of the handover questionnaire was examined in Thompson’s study [10], which indicated good internal consistency of the scale with a Cronbach’s alpha value of 0.93. Furthermore, the current study tested the questionnaire for internal consistency reliability using Cronbach’s alpha, which confirmed reliability with a value of = 0.871.

### Data collection and ethical considerations

Written approval to collect the required data was obtained from the hospital’s managers after obtaining approval from the institutional review board (IRB) of King Abdullah International Medical Research Center (KAIMRC) with approval number SP21J/457/11. With permission from the nurse managers, data were collected from nurses during their breaks as suggested by the nurses. The nurses agreed, and they suggested the researchers return during their break time to fill out the questionnaire without forcing them or interfering with their rights. The questionnaire was distributed individually to CCUs nurses according to their assigned shifts which identified from their time schedule (roster) taken from the nurse managers. The required time to complete the questionnaire was 20 min. Data were collected over two months (March to May 2022). All ethical considerations were maintained and participants were consent forms obtained. They were informed about the study’s purpose and design, and they were assured that their participation was entirely voluntary, that they could opt out at any time and that their data would be treated with strict confidentiality.

### Data analyses

The data were entered the social sciences statistical program IBM SPSS (version 25). The Shapiro‒Wilk test was used to determine the normality of the data. Frequencies and percentages were used to present demographic characteristics and means and standard deviations (SDs) were used to present continuous variables. Pearson correlation coefficient analysis (r) and regression analysis (R^2^) were used to test the nature of the relationship between the studied variables. All statistical analyses were performed using P values of ≤ 0.05 with a 95% confidence interval.

## Results

### Participants characteristics

A total of 201 nurses were participated in this study. Most participants were female nurses (n = 189, 94.0%) and non-Saudi nurses (n = 160, 79.60%). Slightly more than one third of nurses (n = 87, 43.3%) were aged between 31 and 40 years old. Nurses were distributed among the different CCUs and ranged from 7.5% to 22.0. The highest percentage of nurses (n = 122, 60.7%) held a bachelor’s degree. Nurses had a total mean of 11.07 (SD 7.75) years of experience. The highest percentage (n = 94, 46.8%) had from10 to 20 years of experience, followed by (n = 81, 40.3%) with < 10 years of experience. The highest percentages (n = 122, 60.7%) played both receiving and giving handover roles, followed by incoming nurses receiving handover (n = 55, 27.4%). Table 1.

**Table 1:** Demographics and work-related characteristics of nurses working in critical care units (N = 201)

Demographics and work-related characteristics	Total (N = 201)	
	No.	%
**Nationality**		
Saudi	41	20.4
Non-Saudi	161	79.6
**Gender**		
Male	12	6.0
Female	189	94.0
**Age**		
25–30	59	29.4
31–40	87	43.3
41–50	44	21.9
above 50	11	5.5
**Critical Care Units**		
Acute Medical Unit (AMU)	15	7.5
Intensive Care Unit (ICU)	52	25.87
Pediatric Intensive Care Unit (PICU)	27	13.4
Neonatal Intensive Care Unit (NICU)	45	22.4
Pediatric Coronary Intensive Care Unit (PCICU)	22	10.9
Adult Coronary Intensive Care Unit (ACICU)	11	5.5
Adult Critical Coronary Intensive Care Unit (ACCICU)	19	9.5
**Years of experience**		
< 10	81	40.3
10–20	94	46.8
20–30	21	10.4
≥ 30	5	2.5
Min. – Max.	0.08–35.0	
Mean ± SD.	11.07 ± 7.75	
**Education level**		
Diploma	61	30.3
Baccalaureate	122	60.7
Master	18	9.0
**What is your role in shift handover?**		
Outgoing nurses (giving handover)	24	11.9
Incoming nurse (receiving handover)	55	27.4
Varied (both roles).	122	60.7

### Perceived handover quality

Table 2 shows that the highest percentages of nurses reported either good quality of handover (n = 98, 48.8%) or very good (n = 73, 36.3%). Participants were varied in their perception of the staffing factor, considering their shift workload in their units as adequate (38.3%), inadequate (33.3%), or marginal (28.1%). Over half of nurses described their current level of experience as competent (60.7%). The highest percentages of nurses (59.7% and 81.1%) reported experiences of intrusion and distraction, respectively. The main sources of intrusion were families (54.7%), colleagues (49.8%), and patients (47.3%), while the main sources of distraction were alarms from monitors and IV pumps (79.1%) and call bells (43.8%). The negative impact of intrusion and distraction on handover quality was reported by 35.8% and 49.8% of nurses, respectively. Regarding handover tools, Table 2 illustrates that the majority of nurses used an electronic medical record (75.1%) in their handover. Most nurses received handover through face-to-face communication (97.0%), and if handover communication was not face-to-face, it would be delivered in writing (51.2%) and orally (48.8%). Most nurses (98.0%) reported that the last shift handover was guided by a handover tool, mainly a mnemonic ISBAR tool (80.0%).

**Table 2:** Determinants of handover quality as perceived by critical care nurses (N = 201)

Determinants of handover quality	Total (N = 201)	
	No.	%
Handover quality		
Poor	2	1.0
Fair	11	5.5
Good	98	48.8
Very good	73	36.3
Excellent	17	8.5
Staffing		
Inadequate	67	33.3
Marginal	57	28.4
Adequate	77	38.3
Perceived level of current Nurse experience		
Novice	11	5.5
Advanced beginner	17	8.5
Competent	122	60.7
Proficient	20	10.0
Expert	31	15.4
Intrusions experienced		
Yes	120	59.7
No	81	40.3
The impact of intrusion on handover		
Very negative impact	9	4.5
Negative impact	72	35.8
Neutral / no impact	98	48.8
Positive impact	22	10.9
Very positive impact	0	0
Sources of intrusion		
Patients	95	47.3
Families	110	54.7
Colleagues	100	49.8
Other	43	21.4
Experienced distraction		
Yes	163	81.1
No	38	18.9
Distraction impact on handover		
Very negative impact	7	3.4
Negative impact	100	49.8
Neutral / no impact	94	46.8
Positive impact	0	0.0
Very positive impact	0	0.0
Sources of distraction*		
Call bell	88	43.8
Alarms from monitors and iv pumps	159	79.1
Other	69	34.3
Technology used in received handover *		
Electronic medical record	151	75.1
Bedside documentation technology	109	54.2
Handheld applications	39	19.4
Face-to-face communication handover		
Yes	195	97.0
No	6	3.0
If handover communication was not face-to-face, how was it delivered?		
Written	103	51.2
Oral	98	48.8
Shift handover guided by a tool		
Yes	197	98.0
No	4	2.0
Type of tool		
Checklist	15	7.5
Mnemonic device such as SBAR, ISHARED, IPASSTHEBATON	161	80.1
Other	25	12.4

*Multiple response options

### Determinants of handover quality

For the descriptive mean values, the overall mean score for handover quality was average (2.95 ± 0.41). Regarding factors (positive–negative) affecting the overall quality of handover, Table 3 show the highest mean scores were related to focus of attention (3.87 ± 0.84), technology (3.87 ± 0.80), relationships (3.63 ± 0.68), safety climate (3.49 ± 0.53), and cognitive capacity (3.41 ± 1.06). While staffing (2.05 ± 0.85), and time stress (2.37 ± 0.80) had the lowest mean scores.

**Table 3:** Univariate linear regression for factors affecting overall quality of handover

Factors	Mean ± SD	*B*	*β*	R^2^	F	t	p	95% CI	
								LL	UL
Staffing	2.05 ± 0.85	0.226	0.249	0.062	13.185	3.631	< 0.001^*^	0.103	0.349
Intrusions	2.68 ± 0.75	0.203	-0.198	0.039	8.121	2.850	0.005^*^	0.063	0.344
Distractions	2.52 ± 0.62	0.196	-0.158	0.025	5.074	2.253	0.025^*^	0.024	0.368
Cognitive capacity	3.41 ± 1.06	0.007	0.232	0.054	11.371	3.372	0.001^*^	0.003	0.011
Focus of Attention	3.87 ± 0.84	0.014	0.395	0.156	36.856	6.071	< 0.001^*^	0.010	0.019
Anxiety	3.22 ± 0.84	-0.007	-0.182	0.033	6.819	2.611	0.010^*^	-0.012	-0.002
Time stress	2.37 ± 0.80	-0.008	-0.217	0.047	9.860	3.140	0.002^*^	-0.014	-0.003
Time pressure	2.77 ± 0.91	-0.006	-0.167	0.028	5.687	2.385	0.018^*^	-0.010	-0.001
Acute fatigue	2.54 ± 0.65	-0.009	-0.191	0.037	7.563	2.750	0.007^*^	-0.016	-0.003
Chronic Fatigue	2.55 ± 0.91	-0.005	-0.152	0.023	4.682	2.164	0.032^*^	-0.010	0.000
Relationships	3.63 ± 0.68	0.013	0.300	0.090	19.727	4.442	< 0.001^*^	0.008	0.019
Safety climate	3.49 ± 0.53	0.015	0.268	0.072	15.405	3.925	< 0.001^*^	0.008	0.023
Technology	3.87 ± 0.80	0.008	0.218	0.048	9.972	3.158	0.002^*^	0.003	0.014

SD: Standard deviation F,p: f and p values for the model R^2^: regression Coefficient of determination *B = unstandardized regression coefficient, β= standardized regression coefficient, CI = confidence interval*

LL: Lower limit *: Statistically significant at p ≤ 0.05 UL: Upper Limit

In addition, Table 3; Fig. 1 show positive and significant correlations between overall handover quality and each of staffing (*β* = 0.249, R^2^ = 6.2%), cognitive capacity (*β* = 0.232, R^2^ = 5.4%), focus of attention (*β* = 0.395, R^2^ = 15.6%), relationships (*β* = 0.300, R^2^ = 9.0%), safety climate (*β* = 0. 268, R^2^ = 7.2%), and technology (*β* = 0.218, R^2^ = 4.8%), where p < 0.05. All these factors could contribute positively to the prediction of handover quality, where the value of the regression analysis ranged between 5.4% and 15.6%. In contrast, negative correlations were found between overall handover quality and each of intrusions (*β* = -0.198, R^2^ = 3.9%), distractions (*β* = - 0.158, R^2^ = 2.5%), anxiety (*β* = -0.182, R^2^ = 3.3%), time stress (*β* = -0.217, R^2^ = 4.7%), time pressure (*β* = -0.167, R^2^ = 2.8%), acute and chronic fatigue (*β* = -0.191, -0.152 and R^2^ = 3.7, 2.3%) where p < 0.05. All these factors could negatively affect the prediction of handover quality, where the value of the regression analysis ranged between 2.3 and 4.7%.

Regarding barriers and facilitators to nurses’ handover, the number of nurses who answered the open-ended questions about barriers and facilitators that affect the quality of handover from their perspective was 151 (75.2%) out of the total sample (N = 201). All respondents reported shift schedules (night) and length of working hours, inadequate staffing, and staff assignment/workload as the main barriers that affect their handover quality, followed by language and interpersonal communication barriers (66.23%), a lack of knowledge and experience in documentation, and using tools such as SBAR (66.23%). All nurses reported the provision of adequate staffing and training on nurses’ documentation and handover as facilitators for improving nurses’ handover. Also, about two-thirds of nurses (66.23%) reported teamwork, a supportive climate, and continuing the use of a structured handover tool as facilitators. See Table 4 for the responses to open-ended questions.

**Table 4:** Factors affecting the quality of nurses’ handover from nurses’ perspectives (responses to open-ended questions)

Responses	No.	%
Perceived barriers to nurses’ handover * (N = 151)		
1. Shifts schedule (night)/length of working hours	151	100.0
2. Inadequate staffing & staff assignment/workload	151	100.0
3. Language and interpersonal communication barriers (Saudi-non-Saudi)	100	66.23
4. Lack of knowledge and experience in documentation and using tools such as SBAR	100	66.23
5. Poor quality of nurses’ documentation	55	36.4
6. Increased conflict and poor relationship among staff (nurse-nurse)	40	26.5
Perceived facilitators and recommendation for improving nurse’s handover*(N = 151)		
1. Adequate staffing	151	100.0
2. Adequate training on nurse’s documentation& handover	151	100.0
3. Teamwork and supportive climate	100	66.23
4. Continue use of a structured handover tool	100	66.23

*Multiple responses by one participant

Furthermore, the analysis of nurses’ demographics and handover quality showed no significant difference in the perceived handover quality according to nurses’ years of experience, educational level, and nurse’s role in handover. Only gender and nationality showed significant differences, where the mean score among female nurses (3.92 ± 0.79) was higher than that of male nurses (U = 0.777, p = 0.046) and non-Saudi nurses were higher than Saudi nurses (t = 2.591, p = 0.010). See Table 5.

**Table 5:** Handover quality according to nurses’ demographics

Demographics	Overall Hanover quality	Test of sig.	p	
	Mean ± SD.			
**Nationality**				
Saudi	2.80 ± 0.40	t = 2.591	0.010*	
Non-Saudi	2.99 ± 0.41			
**Gender**				
Female	3.92 ± 0.79	U = 0.777	0.046^*^	
Male	3.43 ± 0.76			
**Years of experience**,				
< 10	3.40 ± 0.83	H = 1.922	0.589	
10–20	3.48 ± 0.71			
20–30	3.67 ± 0.80			
≥ 30	3.20 ± 0.45			
**Education level**				
Diploma/ Registered nurse	3.43 ± 0.72	H = 2.511	0.285	
Baccalaureate	3.52 ± 0.74			
Master’s degree	3.17 ± 1.04			
**Role in shift handover**				
Outgoing nurses (giving handover)	3.54 ± 0.51	H = 0.620	0.733	
Incoming nurse (receiving handover)	3.42 ± 0.85			
Varied (both roles).	3.46 ± 0.77			

SD: Standard deviation U: Mann Whitney test H: H for Kruskal Wallis test:

*Statistically significant at p ≤ 0.05

## Discussion

### Perceived handover quality

The quality of nursing handover has become an international priority to ensure patient safety. Our study revealed that the majority of nurses in the current study reported good-quality handover with average mean. These results could be explained by the nature of work in critical care units, which is characterized by multiple and complex care procedures and the instability of patients’ conditions, which entails frequent exchange of patient information. Abou Elaa et al. [21] reported that most studied nurses had a moderate handover degree in CCUs, which is consistent with our findings. Additionally, Manias et al. [22] reported that nurses perceived their own handovers as good, and Thompson [10] reported the mean score for handover quality as average. Liu et al. [23] reported that the handover evaluation of nurses in general hospitals indicated a higher level of handover quality. The variation in the reported levels might be related to many factors that could affect the quality of nurses’ handover in different settings. Discussion of the following determinants may explain the causes of these perceived ratings.

### Determinants of handover quality

This study investigated Saudi CCU nurses’ perspectives on the determinants of handover quality. The findings highlighted three fundamental points linked to handover: (1) handover communication; (2) patient safety; and (3) the role of the nurse. The following discussion will present the determinants in relation to these three points in the form of positive and negative determinants.

#### (1) Handover communication

-***Positive determinants***

Our correlation and regression analysis revealed that cognitive capacity, focus of attention, and relationships are positive predictors and determinants of handover quality that could improve handover communication. Both ***cognitive capacity*** and ***the focus of attention*** factors describe cognitive processing abilities that are required for handover communication and ultimately affect nurses’ ability to communicate clear and correct information as well as receive and store this information based on their understanding and knowledge of a situation and surrounding activity. Our results are parallel with previous studies reporting that interruptions and competing demands during handover may break nurses’ concentration and divert attention, causing them to omit key pieces of information and ultimately leading to incomplete handover and adverse consequences [24, 25]. In contrast, Thompson [10] found that neither cognitive capacity nor focus of attention were significant predictors of handover quality.

The ***relationship*** between the incoming and outgoing nurses was found to be a positively significant predictor of handover quality. This is an interesting and unsurprising finding because incoming and outgoing nurses often have reciprocal relationships that reflect collaboration and group cohesion. Reciprocity and positive relationships can reduce risk because incoming nurses are more likely to ask questions and clarify information with outgoing nurses, which may reduce information omissions and misperceptions. Therefore, developing a spirit of teamwork among unit members is critical for maintaining collaboration and coordination among team members while reducing conflict. Our finding is consistent with other handover-related literature suggesting that positive relationships between members positively influence the quality of handover communication [10, 11, 26]. Additionally, Wang et al. [27] revealed a significant positive correlation between group cohesion and the quality of nursing handovers. In this context, many authors have emphasized the importance of a supportive and respectful work environment that conveys appreciation and gratitude toward nurses’ work and effort as a popular strategy that promotes a positive work relationship and well-being [27–29].

-***Negative determinants***

On the other hand, nurses in the current study, reported ***intrusion*** and ***distraction*** as having a negative impact on the quality of handover and the communication process. Families, coworkers, and patients were the primary causes of intrusion, whereas alarms from monitors, IV pumps and call bells were the primary sources of diversion. This conclusion may be attributable to the fact that intensive care units are complex workplaces where staff members may interrupt and distract each other during handover because something important must be done immediately. Additionally, SMS may be a distraction during handovers, which is unsurprising in this time of ubiquitous mobile phone access. This distraction may result in a failure of communication between health care providers, which may lengthen the handover time and result in the loss or omission of vital information due to interruptions in communication, thereby negatively affecting the quality of handover and putting patient safety at risk. Similar to the present study, numerous nurses in Thompson’s study [10] reported unfavorable intrusion experiences. Our findings are consistent with those of Abou Elaa et al. [21] and Kowitawakul et al. [14], who discovered that nurses in CCUs are frequently interrupted by telephone calls, ventilators, monitor alarms, physicians’ requests, patients’ relatives’ questions, overlap in visiting hours, interrupting or portable ECG machines, and background noise. Although it is difficult to prevent distractions in CCUs, it is essential that all members make active efforts and collaborate to reduce distractions. During handovers, it would be a good idea for a team member to take care of the patient and his or her family.

Additionally, nurses reported in their open-ended responses that ***a lack of knowledge and experience in documentation***, using tools such as ISBAR, and ***poor quality of documentation*** were barriers that affected the quality of their handover. The quality of handover does not rely only on the application of a standardized handover tool; nurses’ communication and documentation skills and their understanding of ISBAR might also be potential factors that predict the quality of handover. If nurses’ documentation is negatively affected, the quality of handover will also be affected. Similarly, Abou Elaa et al. [21] reported that improving the quality of care depends on the quality of documentation. Kowitawakul et al. [14] stated that bedside handover requires accurate documentation and existing supportive materials (e.g., guidance and handover tools) to support communication, prevent information from being lost, and enable staff who were not present at handover to access information. In their systematic review, Raeisi et al. [9] emphasized the importance of using a checklist or a standardized tool in the handover process. Therefore, educating and encouraging all nurses to use tools during handovers might be one strategy for improving the handover process [8], which is in line with our nurses’ recommendations. Additionally, Manias et al. [22] made several recommendations for improvement regarding the use of structured checklists, compliance with standards and procedures, and access to and clarity of information.

#### (2) Patient safety

-***Positive determinants***

Our study found that safety climate, technology and handover tools are among the positive determinants that could affect handover quality and promote patient safety. ***Safety climate*** reflects the observable aspects of the safety culture and includes employees’ perceptions of safety-related policies and practices as well as perceptions of management priorities for safety within the organization [30, 31]. The current study support previous findings that the safety climate influences and predicts handover quality [10, 11]. Research in support of the study findings has identified an association between a positive safety climate and increased staff safety behavior. Moreover, Piper et al. [17] clarified that effective handover can only be enhanced through a supportive climate, organizational support, and structures together with individual health professionals taking responsibility to improve communication networks and processes. Hence, nurse managers should support nurses by promoting a safe work climate and safety culture [31, 32].

Additionally, the availability of handover-related ***technology*** and ***handover tools*** were found to be a significant predictor of handover quality. Most nurses in this study used electronic health records (EHRs) and bedside documentation technology through face-to-face communication. Our hospital and CCUs are fully equipped with a computerized technology system called “Best Care,“ which includes EHRs, laboratory results, medication charts, and radiological investigations, and all patient information is available across different authorized services. Using electronic tools, such as handheld devices and EHRs, could facilitate and improve information transfer at handovers among nurses and medical staff without missing important patient data. This result is consistent with the findings of Methangkool et al. [26], who found that technology and the use of an EHR improve communication among nurses by organizing and streamlining patient information. In contrast, another study found that technology was not a significant predictor of handover quality [10]. We suggest that as technology continues to advance in the form of handheld applications and mobile devices, the technology factor will continue to influence handover communication in the future.

-***Negative determinants***

In contrast, nurses in this study considered the shift ***workload*** in their units inadequate and marginal. Workload may lead to increased pressure to perform, pushing practice beyond the boundaries of safety and having a negative impact on handover and patient safety. This result is also congruent with the qualitative responses of nurses to open-ended questions, which reported nursing shortages, staff assignments, and workload as barriers to handover quality. A Saudi study conducted by Alahmadi and Alharbi [33] found that the growth in demand for nurses, bed capacities, insufficient numbers of individuals joining the nursing profession, overtime shifts, and decreased staffing were all factors that could contribute to nurses’ increased workload and affect patient safety. Additionally, Thompson [10] reported that increased workload negatively influenced the quality of handover, which was related to inadequate staffing levels and a shortage of staff as well as triage flow and time pressure. Therefore, the appropriate and fair distribution of rotational shifts (day–night) and fair work assignments are necessary strategies to manage nurses’ workload [28].

#### (3) The role of the nurse

-***Positive determinants***

Similar to many studies, our findings revealed that standardized handover tools, and staffing can affect the role of nurses in handover. In our setting, shift handover was guided mainly by the ***ISBARQ*** as the basic and standardized communication and handover tool. It is used for structuring handover practices and aids in promoting prioritized presentations and eliciting the essential elements of handovers. Previous research demonstrated how the ISBARQ tool can help nurses in their role to establish logical, structured communication during handovers. In turn, it would lessen the likelihood of information being lost during the handover process, improve communication, and eliminate misunderstandings [2]. Additionally, the application of SBAR/ISBARQ has shown positive effects, such as increased patient satisfaction with the handover style [2, 5, 18] and increased patient safety outcomes, such as a reduction in patients’ adverse events and complications [2]. Given that nurses have different training, communication skills, and ideas about how things are going, simulation could be a way to standardize electronic communication during the handover process [26]. More research is needed into the potential benefits of using technology during handovers.

Although ***staffing*** was among the positive factors that could influence handover quality in this study, nurses attributed the lowest mean scores of the handover dimensions to staffing. Similarly, Piper et al. [17] commented on staffing issues within the work environment and believed that there was a direct relationship between underreporting of incidents and staffing problems and problems with poor handover. These findings are also congruent with and supported by nurses’ reports in the qualitative section, namely, that the provision of adequate staffing and training on nurses’ documentation and handover, teamwork, a supportive climate, and continuing the use of a structured handover tool are facilitators and recommendations for improving nurses’ handover quality.

-***Negative determinants***

The present study showed that psychological precursors, including anxiety, time stress, and pressure, in addition to acute and chronic fatigue factors, may negatively affect the prediction of handover quality and the role of the nurses. Moreover, the responses of nurses to open-ended questions identified 12-hour shift schedules, fatigue from long working hours, and nurses’ workload as barriers to effective handover.

***Psychological precursors*** can lead to hindered communication and impaired performance and are related to nurses’ well-being and fatigue level, so they are assumed to influence handover quality. Nurses are likely to experience time stress and pressure in CCUs because they often have several tasks, such as charting to complete before the end of their shifts, so they might become anxious and worry about completing the required tasks. Additionally, perceived ***fatigue*** levels may be attributed to nurses’ workload with rotational shift arrangements as the hospital currently adopts 12-hour shift rotations. Fatigue can result in decreased mental acuity and vigilance, both of which are believed to negatively influence incoming nurses’ ability to receive handover communication.

Similarly, Alsayed et al. [28] reported that nurses’ shift work, time pressure, and demanding work schedules can be stressors that affect their physical and psychological health and well-being, frequently inducing fatigue and subsequently influencing their work performance and thus impacting patient safety and the quality of care. Nurses’ fatigue results in mood changes, reduced mental acuity, social problems, degradation in performance and work capability, physical pain, greater risk of needle stick injuries and musculoskeletal disorders, and medication errors [28, 33, 34]. Therefore, stress management and fatigue mitigation programs are essential to promote nurses’ cognitive and psychological well-being. In contrast, Thompson [10] found that acute or chronic fatigue, anxiety, and time stress were not significant predictors of handover quality. It is suggested that the effects of psychological precursors on handover quality should be further explored using observational and qualitative approaches.

Other factors reported by the nurses on open-ended questions that could affect handover communication, patient safety and the role of the nurses are ***language and interpersonal communication barriers*** (Saudi/non-Saudi) and ***conflict and poor relationships among staff*** (nurse‒nurse, nurse‒physician). These results could be attributed to the nursing workforce in Saudi Arabia, where nursing is dominated by expatriate non-Saudi nurses from various countries, which creates challenges linked to cultural, language, and religious differences and communication barriers. Previous studies have explained that there are differences in language, religion, and culture among nurses who provide health services in Saudi Arabia that can directly influence relationships and work communication [20, 35]. Although this factor is a challenge in the Saudi health context, several strategies could be applied to improve communication and relationships in the unit. One of the strategies that has been applied recently in our hospital is the assignment of a team member as a unit assistant who helps with translation and facilitates communication in the units. Additionally, the provision of soft skills and communication training programs would be helpful.

Finally, other individual factors could be a part of the determinants of handover quality, where the current study revealed a significant difference in the perceived overall handover quality according to nurses’ nationality (Saudi and non-Saudi) and gender. Non-Saudi and female nurses showed a higher mean than Saudi and male nurses. This could be due to the different social and gender role expectations for women and men and the dominance of female and expatriate nurses in the hospital nursing work force. On the other hand, the results showed no significant differences in the perceived handover quality according to nurses’ age, years of experience, educational level, or role in handover. Ghosh et al. [36] found that female nurses perceived handovers in a more positive way than male nurses, and there were no significant differences among the nurses’ scores in terms of age, level of education, experience, and overall nursing handover score. On the other hand, Nagammal et al. [37] found no significant difference between nurses’ views on patient handover and gender. Therefore, we could suggest that cultural background is influential on the success of handover in terms of developing perceptions of the handover process, however, it needs further investigation.

#### Strengths and limitations

The present study has some strengths and limitations. This study is one of the first efforts to identify determinants that influence the quality of nurse-to-nurse shift handover in Saudi critical care units from nurses’ perspectives, which could open a path for future replicated and in-depth qualitative studies. Nevertheless, this study has some limitations. First, due to the descriptive research design, we were unable to establish causality between the determinants of nursing handover quality. Second, all data were obtained from self-report questionnaires; thus, reporting bias cannot be avoided. Third, the inclusion of only a single hospital’s nurses in the survey may limit the generalizability of the findings. Further studies that recruit participants from wider areas are needed to verify the results of the current research. Additionally, our research included a certain number of variables, which might provide a fragment of the picture in terms of the quality of handover. More potential factors that may lead to successful handover should be explored in further studies.

## Conclusion

Nursing handover is a multidimensional phenomenon that is particularly challenging in critical care units. Our findings presented many determinants that could affect the quality of handover and, in turn, affect three important points linked to shift handover, including communication efficacy, patient safety, and the role of the nurse in their handover. Through an understanding of the determinants that contribute to or hinder handover quality, it is possible to develop targeted interventions aimed at improving communication and the quality of shift handover in CCUs. The current study concluded that nurses reported good handover quality. The correlation and regression analyses illustrate that staffing, cognitive capacity, focus of attention, relationships, and safety climate factors could contribute positively to the prediction of handover quality. In contrast, intrusions, distractions, anxiety, time stress, time pressure, and acute and chronic fatigue factors could negatively affect the prediction of handover quality. The qualitative data analysis of open questions identified shift schedules (night) and length of working hours, inadequate staffing and staff assignment/workload, language barriers, a lack of knowledge and experience in documentation, and increased conflict and poor relationships among staff as the main barriers that affect handover. On the other hand, nurses reported the provision of adequate staffing and training on nurses’ documentation and handover, teamwork, a supportive climate, and the continuing use of structured handover types of shifts as facilitators for improving nurses’ handover quality.

### Recommendation and implications of the study

***Implication for practice***.

- Our findings highlighted in the discussion the need for a comprehensive approach to improving handover quality. Generally speaking, hospital managers should promote handover standardization and training, safety climate, organizational support, creative workforce planning, flexible work schedules, and fatigue management as vital strategies to improve nurses’ well-being, communication efficacy, the role of nurses, which impact the quality of handover and promote patient safety. Therefore:
- Hospital managers have to invest in and implement institution-wide standardized handover training programs to improve handover practice, control disruption, reduce errors, and reduce communication failure. Also, regular units’ staff meetings should be held, and when necessary, corrective and preventive measures should be put in place immediately.
- Hospital and nurse managers have to develop and follow clear policies and guidelines that regulate all units’ activities, including handover policies such as handover tools, physician rounds, visiting hours, handling telephone messages, and all CCU routine care, to decrease the interruptions nurses face during their work. These policies should be documented and communicated to staff nurses during their orientation and job induction, with appropriate supervision of their use.
- Hospital administrators and unit managers share the responsibility to promote a safer and more supportive work environment for all. They should be aware of how psychological precursors and fatigue affect the quality of handover and nurses’ and patients’ outcomes. Therefore, stress and conflict management, fatigue prevention, and healthy lifestyle habits are strategies that would benefit administrators, nurse managers, and clinical nurses.
- Nurse managers could allow nurses to handle their work on a more flexible schedule, which could reduce stress and pressure related to time and make it easier to reward good handover practices.

***Implication for future research***.

- Future research and strategy development must focus on the handover process, and the role and practice of electronic media in communication must be considered. Longitudinal research is needed to investigate the causal linkages among the factors that contribute to quality handover. We recommend conducting an interventional study on standardized handover to investigate its effect on nurses’ handover quality. A qualitative study is recommended to obtain a more in-depth understanding of associated factors, barriers, and facilitators to nursing documentation and handover. Further research will need to take patient and family experiences into account to understand what an effective handover would look like for them. Also, we recommend comparing patient outcomes from handovers conducted on units with good and poor practice environments. Additionally, further studies should be conducted to examine whether other personal and contextual factors (e.g., organizational support, work pressure, self-efficacy, and burnout) affect the quality of nursing handovers.

## Data Availability

All data generated or analyzed during this study are included in this published article [and its supplementary information files].

## Declarations

### Approval confirmation

All authors have approved the manuscript for submission.

### Content of the manuscript

We confirm that the content of the manuscript has not been published or submitted for publication elsewhere.

## Abbreviations

AHRQ: Agency for Healthcare Research and Quality
AMA: Australian Medical Association
ANOVA: Analysis of Variance
CCUs: Critical care units
CI: Confidence interval
EHRs: Electronic Health Records
IRB: Institutional Review Board
ISBARQ: Introduction, situation, background, assessment, recommendation, questions, and feedback
JCI: Joint Commission International
KAIMRC: King Abdullah International Medical Research Center
KKH: King Khalid Hospital
KAMC-J: King Abdul-Aziz Medical City-Jeddah
KSAU-HS: King Saud bin Abdelaziz University for Health Sciences
NCEC: National Clinical Effectiveness Committee
SPSS: Statistical Package of Social Sciences
SBAR: Situation, background, assessment, and recommendation
WHO: World Health Organization
